# Early Diagnosis and Real-Time Monitoring of Regional Lung Function Changes to Prevent Chronic Obstructive Pulmonary Disease Progression to Severe Emphysema

**DOI:** 10.3390/jcm10245811

**Published:** 2021-12-12

**Authors:** Tony Jung, Neeraj Vij

**Affiliations:** 1 Department of Pediatrics and Pulmonary Medicine, Johns Hopkins University School of Medicine, Baltimore, MD 21287, USA; tjung8@jhu.edu; 2 PRECISION THERANOSTICS INC, Baltimore, MD 21202, USA; 3 VIJ BIOTECH, Baltimore, MD 21202, USA

**Keywords:** chronic obstructive pulmonary disease, COPD, emphysema, lung, point of care diagnostics, regional lung function, oscillation techniques, tomography

## Abstract

First- and second-hand exposure to smoke or air pollutants is the primary cause of chronic obstructive pulmonary disease (COPD) pathogenesis, where genetic and age-related factors predispose the subject to the initiation and progression of obstructive lung disease. Briefly, airway inflammation, specifically bronchitis, initiates the lung disease, leading to difficulty in breathing (dyspnea) and coughing as initial symptoms, followed by air trapping and inhibition of the flow of air into the lungs due to damage to the alveoli (emphysema). In addition, mucus obstruction and impaired lung clearance mechanisms lead to recurring acute exacerbations causing progressive decline in lung function, eventually requiring lung transplant and other lifesaving interventions to prevent mortality. It is noteworthy that COPD is much more common in the population than currently diagnosed, as only 16 million adult Americans were reported to be diagnosed with COPD as of 2018, although an additional 14 million American adults were estimated to be suffering from COPD but undiagnosed by the current standard of care (SOC) diagnostic, namely the spirometry-based pulmonary function test (PFT). Thus, the main issue driving the adverse disease outcome and significant mortality for COPD is lack of timely diagnosis in the early stages of the disease. The current treatment regime for COPD emphysema is most effective when implemented early, on COPD onset, where alleviating symptoms and exacerbations with timely intervention(s) can prevent steep lung function decline(s) and disease progression to severe emphysema. Therefore, the key to efficiently combatting COPD relies on early detection. Thus, it is important to detect early regional pulmonary function and structural changes to monitor modest disease progression for implementing timely interventions and effectively eliminating emphysema progression. Currently, COPD diagnosis involves using techniques such as COPD screening questionnaires, PFT, arterial blood gas analysis, and/or lung imaging, but these modalities are limited in their capability for early diagnosis and real-time disease monitoring of regional lung function changes. Hence, promising emerging techniques, such as X-ray phase contrast, photoacoustic tomography, ultrasound computed tomography, electrical impedance tomography, the forced oscillation technique, and the impulse oscillometry system powered by robust artificial intelligence and machine learning analysis capability are emerging as novel solutions for early detection and real time monitoring of COPD progression for timely intervention. We discuss here the scope, risks, and limitations of current SOC and emerging COPD diagnostics, with perspective on novel diagnostics providing real time regional lung function monitoring, and predicting exacerbation and/or disease onset for prognosis-based timely intervention(s) to limit COPD–emphysema progression.

## 1. Introduction

Chronic obstructive pulmonary disease (COPD) is characterized by airway obstruction and airflow limitation caused by damage to the alveoli (emphysema) that is worsened by recurring episodes of infections or acute exacerbations (AE-COPD). AE-COPD amplifies inherent inflammatory–oxidative stress responses and impairs airway defense and repair mechanisms, leading to the progressive decline in lung function [1,2,3]. Briefly, inflammation of the airway (bronchitis), which is influenced by environmental, age-related, and/or genetic risk factors, initiates COPD pathogenesis and emphysema progression. There are varying levels of COPD severity that are classified as Global Initiative for Chronic Obstructive Lung Disease (GOLD) stages based on the extent of emphysema and lung function impairment [1]. The primary risk factor for COPD pathogenesis is exposure to harmful particles or gases, such as those found in cigarette or biomass smoke. Thus, cigarette smokers are known to have COPD-related respiratory dysfunctions, limited airflow, and higher prevalence of death [4,5,6]. Similarly, harmful dusts, chemicals, and air pollutants present in the environment or biomass smoke have also been found to increase inflammation of the airways [7,8,9], where repeated exposure can initiate emphysema. Additionally, comorbidities can further accelerate COPD pathogenesis. As an example, those with a history of asthma were found to have a 10- to 30-fold increased risk of developing COPD [10].

Moreover, hereditary factors, such as alpha-1 antitrypsin deficiency (AATD), found in approximately 5% of subjects with COPD, predisposes the subject to develop emphysema. Briefly, alpha-1 antitrypsin is a serine protease inhibitor primarily produced in the liver to protect tissues from damage due to infection and resulting inflammation, where the absence of AAT and/or presence of recurring respiratory exacerbations in these subjects initiates lung tissue damage due to underlying inflammation, leading to COPD-emphysema pathogenesis [1,11]. It is noteworthy that all subjects with AATD do not develop COPD, highlighting the requirement of a trigger such as first- or second-hand smoke, infection, other toxic/inflammatory agents, etc. Additionally, while studying COPD from an epidemiological perspective, the Boston Early-Onset COPD study demonstrated that smokers that are first-degree relatives of subjects with severe, early-onset COPD had a threefold risk of airflow limitation compared to smokers without family history of COPD [12], verifying the role of genetic predisposition in COPD pathogenesis. Furthermore, deficiencies in lung growth or development are also linked to increased exposure to environmental and/or genetic risk factors, specifically, individuals who have reduced maximal attained lung function have limited airflow, leading to the development of COPD over time.

Moreover, COPD is an irreversible obstructive lung disease that is often diagnosed late. In contrast, asthma is a reversible obstructive lung disease that is generally identified early, allowing timely intervention. Even though COPD can be treated to prevent or dampen symptoms and exacerbations, it is a progressive lung disease, where repeated or recurring episodes of acute exacerbations lead to steep lung function decline over time with age to severe and fatal emphysema stages, necessitating lung transplant to prevent fatal outcomes [13,14,15]. Thus, to cease COPD progression to the fatal stages, it is critical that treatment is implemented early, prior to significant advancement of COPD to severe emphysema. This necessitates the need for diagnostic and real-time lung health monitoring procedures that can identify early changes in at-risk subjects’ lungs, prior to progression to severe emphysema stages. Unfortunately, current standards of care (SOC) diagnostics are ill-equipped to accomplish this goal. As of 2018, 16 million US adults were diagnosed with COPD, while greater than 14 million American adults were estimated to remain pulmonary function test (PFT)-undiagnosed using the current gold SOC diagnostic. Thus, due to the lack of appropriate diagnostics for early detection of COPD, the status of subjects’ lung conditions cannot be monitored for timely treatment, leading to severe disease progression that otherwise could have been prevented with early intervention.

The go-to current gold SOC for clinical diagnosis of COPD remains spirometry-based PFT. Briefly, as per current SOC, to be diagnosed with COPD, one must score less than 0.70 on the ratio of forced expiratory volume in the first one second of breath (FEV_1_) to forced vital capacity (FVC) [1]. However, in many cases, this diagnostic tool by itself is not sufficient in accurately predicting COPD presence, especially in the early stages of the disease. This can partially be attributed to the inability of spirometry/PFT to recognize varying severities of the disease, as seen in the Obstructive Lung Disease Northern Sweden Study [16], which found that 50% of those with severe COPD received a clinical diagnosis through spirometry, while only 19% of those with mild COPD received a clinical diagnosis through spirometry, prompting the need for more comprehensive screening procedures and highlighting the unmet need for a diagnostic capable of identifying early subtle or regional changes in lung function. Briefly, spirometry/PFT measures total lung function changes, and it misses regional or early-stage subtle changes in lung function. However, there are other diagnostics that have been used to acutely screen and further validate the presence of COPD, including diffusing capacity for carbon monoxide (DLCO)/arterial blood gas (ABG) analysis and lung imaging modalities, such as X-rays, computer tomography (CT) scans, and magnetic resonance imaging (MRI), but most of these tests used alone or in combination miss early-stage disease. Screening tests, such as the six-minute walk test (6MWT) and COPD questionnaires, although useful in finding indicators of COPD prior to clinical diagnosis and/or monitoring the status of COPD progression, are not robust quantitative measures for timely diagnosis of subtle changes in lung function.

Thus, despite the usefulness of traditional diagnostic tools, there are many weaknesses and critical limitations that need to be addressed as summarized for comparative analysis in Table 1. As discussed above, one of the main limitations of current SOC diagnostics is early detection of the disease. Another significant deficiency of current SOC diagnostics is that they lack the ability to monitor regional changes in the airway as they primarily focus on quantifying global lung function changes. Lung imaging modalities such as CT, MRI, X-ray phase contrast, photoacoustic tomography (PAT), etc., do allow one to observe regional differences or structural changes in the lung, but this comes at the cost of exposure to harmful radiation or toxicity, ruling out its utilization for routine and real-time monitoring and/or intervention. Furthermore, classical imaging modalities provide input on lung function by drawing inferences from structural changes instead of directly quantifying changes in pulmonary function. However, other novel diagnostic tools are emerging to compensate for these shortcomings. As an example, the forced oscillation technique (FOT) measures respiratory function using pressure oscillations, while the impulse oscillometry system (IOS), a commonly used forced oscillation technique, is capable of diagnosing COPD by quantifying the lung’s response to different pressure frequencies [17]. However, this necessitates the significant need for application of artificial intelligence (AI) to analyze audio data from FOT/IOS to automate and further refine these diagnostics to precisely detect COPD [18]. Likewise, other safer, novel lung imaging modalities, such as ultrasound computed tomography (UCT), are also being developed as a healthier alternative, although they will require resolving signal to noise ratio limitations for increased resolution. In addition, electrical impedance tomography (EIT) is unique in its ability to non-invasively quantify changes in regional lung function by 3D reconstruction and analysis of electrical impedance data.

## 2. COPD Screening Tests for Evaluating Disease Initiation and Progression

### 2.1. COPD Screening Questionnaires

Multiple methods are used to screen and monitor for symptoms and indications of COPD pathogenesis and progression. One of the most common is the COPD questionnaire that is routinely used for both initial diagnosis and monitoring of respiratory exacerbations. However, there are multiple adaptations of COPD questionnaires available to evaluate the degree of severity by which COPD affects quality of life (QoL) of the subject and/or disease progression. As an example, the Chronic Respiratory Questionnaire (CRQ) and the St. George’s Respiratory Questionnaire (SGRQ) are extremely comprehensive for detailed screening. However, they are lengthy and complicated for routine use [1,19,20]. In contrast, the Clinical COPD questionnaire (CCQ) and the COPD Assessment Test (CAT) are shorter tests that can be completed in approximately 2 min with high degrees of reliability and validation, providing quantitative input for COPD diagnosis and monitoring of disease progression. Both tests are recommended by the GOLD and are also consistently responsive with interventions [21,22,23], allowing preliminary evaluation of therapeutic efficacy in addition to its application as a screening and monitoring diagnostic tool.

Moreover, direct comparison of the two questionnaires found CAT to be a better predictor of future exacerbation events than CCQ. CAT also has a clinically set threshold for severity of symptoms, while CCQ has a broad range of scores between 1.0 and 1.5, implying that CAT is more suitable than CCQ in the clinical setting where a strict threshold is required [24]. While COPD questionnaires are helpful, they are generally limited in their ability to quantify or predict structural and/or functional changes in the lung. Briefly, these questionnaires measure the overall status of the patient’s respiratory health, but they cannot directly detect or predict changes in lung function or structural defects, hence limiting their utility as an adjunct test supporting functional or prognostic diagnostics.

### 2.2. Six Minute Walk Test

Another routine method of early COPD screening is the 6MWT that can be performed at home, point of care (POC), or the clinic. According to the American Thoracic Society (ATS), 6MWT should be done indoors on a long, flat, straight surface 30 m in length with the distance marked every 3 m and cones placed as turnaround checkpoints at each end. Prior to the test, the patient rests in a chair for about 10 min and then walks up and down the corridor around the cones for 6 min while the number of laps walked are recorded. If feasible, a pulse oximeter can be incorporated to measure heart rate and oxygen saturation (SpO_2_). A readout of decreased walking distance by the subject is not only indicative of the presence of COPD but also its severity. A study showed that a 6MWT distance of less than 350 m indicated a significantly elevated risk of death and hospitalization [25,26,27], showing 6MWT’s utility as a predictor of morbidity and mortality. In addition, 6MWT has an added utility as a predictor of recurring respiratory exacerbations, which are significant contributors of lung function decline. As an example, a two year study performed in Brazil found that patients who scored less than 80% of the predicted value of the 6MWT were twice as likely to have recurring exacerbations [28].

However, the 6MWT has certain limitations, such as that results can be influenced by the pathophysiological characteristics of the individual subject that may or may not be related to COPD. For example, gender, age, sex, weight, and ethnicity can all determine an individual’s ability to exercise over the extended periods of time. Therefore, while a low 6MWT distance correlates with COPD, it does not validate COPD as the cause [29], requiring further evaluation by current SOC diagnostics, such as spirometry-based PFT.

## 3. Current COPD Standard of Care Diagnostics

### 3.1. Spirometry-Based Pulmonary Function Tests for Monitoring COPD Lung Function

Classical spirometry is the widely accepted SOC for clinical diagnosis of chronic COPD and is the most common PFT. The key advantage of the test is limited risk to the subject while providing an objective outcome measure for quantifying changes in total lung function for COPD diagnosis. Briefly, spirometry assesses lung function through inhalation and exhalation breathing maneuvers, requiring significant patient compliance. These measurements are recorded at the maximal point of inspiration, where spirometry can quantify changes in FVC and FEV_1_ of breath to evaluate the subject’s lung function. If the FEV_1_/FVC ratio for the subject is less than 0.70, the diagnosis is COPD positive. Additionally, FEV_1_ also provides a method for classifying varying levels of COPD severities [30]. According to GOLD, if the FEV_1_ is greater than 80%, the COPD subject is classified as GOLD I with mild emphysema, while if FEV_1_ is less than 80% but greater than 50%, the subject is classified as GOLD II with moderate emphysema. Moreover, subjects with FEV_1_ less than 50% but greater than 30% are classified as GOLD III stage COPD with severe emphysema, and those with less than 30% FEV_1_ are classified as GOLD IV stage with very-severe emphysema [1].

Despite the benefits of spirometry in staging and clinical diagnosis of COPD–emphysema, it is limited in its efficacy as well as potential in early and comprehensive diagnosis or monitoring of subtle or regional lung function changes. As seen in the Obstructive Lung Disease Northern Sweden Study discussed above, 50% of subjects with severe COPD received a clinical diagnosis through spirometry, while “only” 19% of those with mild COPD received a similar clinical diagnosis through spirometry, indicating that while spirometry is more adequate in identifying severe COPD, diagnosis becomes more unreliable when the FEV_1_/FVC ratio is within the moderate or mild range [31,32,33]. In addition, other diseases with airflow limitations, such as asthma, must be ruled out [34], although it is feasible to differentiate COPD from asthma on a PFT by using a bronchodilator, as asthma is a reversible lung condition while COPD is irreversible. One of the most significant limitations of spirometry is that while it quantifies changes in total lung function, it is not capable of identifying or quantifying changes in regional lung function, thus completely missing on early subtle changes. Since COPD is a patchy lung disease that originates with regional air flow limitation, PFT does not provide robust qualitative and quantitative data to assess initiation and progression of early-stage disease for timely intervention.

### 3.2. Diffusing Capacity for Carbon Monoxide and Arterial Blood Gas Analysis

The DLCO test quantifies the lung’s efficacy in transporting oxygen from inspired air into the blood cells by measuring the uptake of carbon monoxide (CO) per unit time per mm of driving pressure to evaluate the global lung function. DLCO assesses uptake of CO instead of oxygen (O_2_) because CO’s ability to bind to hemoglobin is many times stronger, thus allowing detection of subtle functional changes. Briefly, the test is performed by having the subject inhale test gas containing CO, tracer gas, O_2_, and nitrogen (N_2_) for 10 s before exhaling, where the exhaled gas is analyzed for CO and tracer gas concentration using a DLCO analyzer. Test results for DLCO are normal at greater than 75% of predicted gas concentration, mild at ~60%, moderate between 40 and 60%, and severe at less than 40% [35,36]. Additionally, since DLCO is measured at full inflation, it also assesses total lung capacity, similar to spirometry, making it an appropriate diagnostic tool for quantifying alveolar volume and, therefore, emphysema [37,38,39].

Thus, even though DLCO is sufficient for quantifying total lung function, it is limited in its ability to distinguish local or regional lung function changes because it comprehensively quantifies the input and the output of gas through exhalation and only provides cumulative data on how efficient the subject’s lung is at absorbing CO. Consequentially, this provides a general outcome measure of global lung function changes but fails to identify specific regional changes, deficiencies, or damages in the lung and thus is obviously incapable of identifying early stage disease.

Similar to DLCO, the ABG analysis test also quantifies the lung’s ability to uptake O_2_ but accomplishes this by quantifying the following: (1) the partial pressure of O_2_ (PaO_2_), which indicates if enough O_2_ is transferred to the blood stream; (2) the partial pressure of CO_2_ (PaCO_2_), which quantifies if enough CO_2_ is transferred out of the blood stream; (3) the pH, which reveals the presence of high CO_2_ levels; and (4) the saturation of O_2_ (SpO_2_), which also evaluates if enough O_2_ is present in the arterial blood stream. Briefly, pH can quantify CO_2_ levels because CO_2_ reacts with water (H_2_O) to form carbonic acid (H_2_CO_3_). If CO_2_ transfer out of the blood stream is insufficient, the accumulation of CO_2_ will lead to a buildup of H_2_CO_3_, noticeably lowering the pH of the blood [40,41]. Because COPD limits airflow, subjects with COPD are expected to have decreased PaO_2_ levels, increased PaCO_2_ levels, decreased pH levels, and decreased SpO_2_ levels relative to a person with healthy lungs, providing a method for objective qualification of global changes in pulmonary function. Additionally, ABG tests are a standard for prescribing supplemental oxygen to COPD patients that have a PaO_2_ value less than 55 mmHg, a SpO_2_ value less than 88%, or a PaO_2_ value between 55 and 60 mmHg for subjects with right heart failure or erythrocytosis [1,42].

Like previously discussed SOC diagnostics, ABG is limited in its capability and only provides a prediction of changes in global lung function by analyzing the composition of arterial blood gas and is incapable of quantifying local regional lung function. Another limitation is that ABG analysis is not indicative of the severity of COPD and is not adequate or efficient for diagnosis of early stages of the disease, limiting its efficacy as a robust monitoring and diagnostic tool for emphysema initiation and progression [43].

### 3.3. Functional Lung Imaging Modalities for Evaluating COPD Progression

Lung imaging modalities are useful diagnostic tools for assessing structural changes and drawing inferences in relation to functional changes to predict COPD pathogenesis and progression. As an example, CT scan is one of the most commonly used modality for lung imaging that harness the penetrating properties of radiation to generate high-resolution tomographic images. During the scan, the subject is placed between a source of X-rays and X-ray detectors, which measure the attenuation of X-ray signals through tissues as they are rotated around the area of interest. However, due to high radiation exposure risks, CT scans are not routinely used for COPD monitoring and diagnosis in accordance with the GOLD guidelines and thus CTs are only utilized for bronchiectasis and lung cancer detection in high “at-risk” COPD subjects [1,44], even though advances in resolution of low X-ray dose CT scans have shown potential application in evaluating COPD because of their ability to specifically quantify and localize artefacts such as emphysema related structural damage(s) by visualizing distinguishably low tissue-density areas surrounded by normal lung tissue to quantify changes in airway and pulmonary vasculature [45,46,47]. Thus, CT scans can help determine the cause of respiratory symptoms as they can view characteristics of regions of lung damage, bolstering their efficacy in specifying information for interventions [48,49], although with limited capability for real time or routine disease monitoring for bedside intervention(s).

In addition, chest X-rays are another classical and widely available diagnostic technique used to image the lungs by transmitting X-rays onto a target region, where they penetrate through the body, and data are recorded by a detector at the other end. The attenuation of X-rays after they pass through the target region are compared to the initially applied radiation to generate an image. Typically, X-rays are not useful in COPD diagnosis and can only detect COPD when it is severe, limiting its scope for monitoring and diagnosis. Instead, X-rays are used to exclude other causes that may be inflicting respiratory issues, such as comorbidities, or to visualize enlarged lungs, etc. [1].

MRI is another example of an imaging modality that utilizes magnetic fields and radio waves instead of ionizing X-ray radiation to generate images of the tissue of interest. Historically, MRIs have not been widely used in lung imaging and COPD diagnosis due to the low proton density in lung tissue and rapid signal decay caused by artefacts [50]; however, as technology continues to advance, MRIs show potential in competing with established lung imaging modalities as an emerging COPD diagnostic. For example, with recent development, lung function can be quantitatively evaluated from MRI by introducing hyperpolarized gases or contrast agents to the subject’s airway, allowing for high resolution visualization of ventilation (V), perfusion (Q), and/or airflow changes [51]. The advantage of this approach is that it is actually measuring V/Q changes instead of drawing inferences from structural changes on an image.

Thus, even though advances in functional analysis software now allow evaluation of regional lung function changes using CT data, it not only lacks real time assessment/capability due to lack of bedside equipment and high risk of radiation exposure, but also does not directly measure changes in lung function as software’s output data are simply a prediction or inference of a structural change rather than direct quantification of the regional or global lung function [48]. Additionally, X-ray imaging and CT scans both utilize ionizing X-ray radiation, which is classified as a carcinogen. Because there is a direct correlation between X-ray exposure and cancer development [52,53], constant use of these modalities for real-time monitoring of chronic lung conditions is limited due to the severe health implications for chronically ill patients. Lower dose radiation CTs are being developed as a healthier alternative, but this comes at the cost of decreased resolution, limiting its monitoring and diagnostic capabilities. In contrast, MRIs do not utilize radiation, but they require hyperpolarized gases or contrast agents to capture changes in V and/or Q due to the scarce proton density in the lungs. Since these agents can be toxic and cause side effects [54,55], the use of MRI for routine monitoring of disease progression is limited as it cannot be used repeatedly at short intervals. Furthermore, these imaging modalities require complex medical devices that are expensive and available only in hospitals and clinics with radiology units. In addition, these modalities cannot support bedside or real-time monitoring of patients’ lung condition.

## 4. Emerging COPD Diagnostics for Real Time Lung Function Assessment

**Figure 1:**
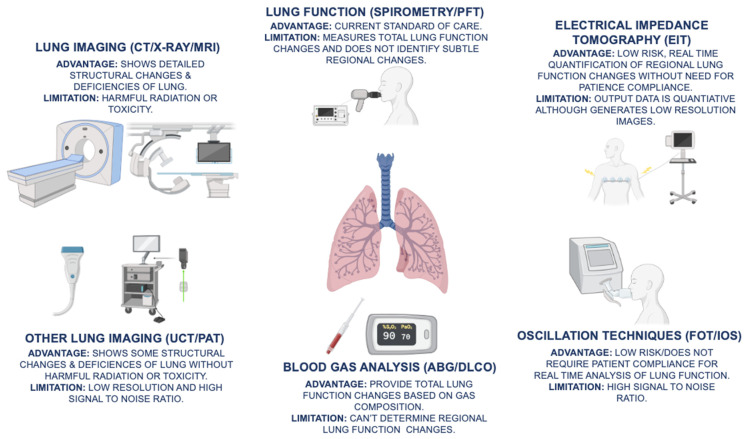
The advantages and limitations of current COPD (chronic obstructive pulmonary disease) diagnostics, which include spirometry/PFT (pulmonary function testing), ABG (arterial blood gas), DLCO (diffusing capacity for carbon monoxide), and lung imaging modalities, such as CT (computed tomography), X-ray, and MRI (magnetic resonance imaging), are illustrated. In addition, emerging novel diagnostic techniques, such as EIT (electrical impedance tomography), FOT (forced oscillation technique), IOS (impulse oscillometry system), UCT (ultrasound computed tomography), and PAT (photoacoustic tomography), provide non-invasive, real-time assessment of changes in lung function.

Significant strides in COPD diagnosis have been made with emerging novel diagnostics capable of early diagnosis and/or real time assessment of disease progression. These techniques include FOT/IOS, PAT, XPC, UCT, and EIT, each with unique advantage’s and/or limitations as shown in Figure 1. The corresponding artificial intelligence (AI) and validation software have also been developed as add-on utilities to achieve robustness and automation for overcoming current limitations for clinical bedside translation.

### 4.1. X-ray Phase Contrast Imaging and Tomography for Functional Lung Imaging

X-ray phase contrast (XPC) utilizes the diffraction of the X-rays to measure the shift in the phase after passing through the tissue to capture weak X-ray absorbing properties that are not detected through conventional X-ray imaging [56], thus showing promise as a novel diagnostic tool, similar to low dose CT. Briefly, due to the relatively high refractive index difference between lung tissue and air, XPC is a powerful tool for capturing details of the lung that may indicate the presence of COPD or other lung defects. As an example, a preliminary clinical study was able to successfully distinguish mild and severe emphysema in mice utilizing an analyzer-based XPC to map airway regions while retaining a sensitivity of 0.80 and a specificity of 0.89, showing its accuracy and potential application in pulmonary diagnostic processes that requires significant clinical development. Additionally, XPC can measure lung air volume capacity by capturing dynamic images of the lung, thus allowing observation of lung function changes at both the regional and global levels [57,58,59]. Briefly, there are five main categories of XPC imaging: (1) propagation-based imaging, (2) analyzer-based imaging, (3) interferometric methods using crystals, (4) grating interferometric, and (5) grating non-interferometric techniques. Each have their advantages and disadvantages, but all depend on X-ray detector resolution, image reconstruction algorithms, X-ray energy, and X-ray divergence for efficient result acquisition [60,61]. The specific advantages of different XPC imaging categories need to be developed in a clinical setting to ascertain the optimal method for capturing specific details and functions of the lung consistent with COPD. In spite of AI or algorithm-based analysis capabilities, resolution and radiation risk remain significant limitations for XPC’s clinical implementation. Moreover, this technique requires complex radiology equipment with limited scope in real-time, or routine bedside monitoring of COPD and respiratory disease progression for timely intervention.

### 4.2. Force and Impulse Oscillometry Measurements for Lung Function Analysis

As discussed above and previously [62], there is a significant unmet need for COPD diagnostic techniques that notably quantify real-time changes in lung function to detect COPD in its early stages for timely intervention. An example of such emerging COPD diagnostic is FOT, which noninvasively assesses lung function by measuring the lung’s response to applied pressure oscillations over normal breathing patterns and quantifies it as impedance, which is the resistance of a system airflow and is calculated from the pressure and airflow recorded throughout the measurement [63,64]. Similarly, IOS is a variation of FOT, where FOT transmits frequencies sequentially, while IOS transmits frequencies as an impulse that can be differentiated into varying frequencies, making the testing process faster while improving the signal-to-noise ratio [65,66], which is the most significant limitation of this technique. The impedance from these tests represents the responsiveness of the respiratory system, making FOT/IOS a significant prospective diagnostic tool for quantifying bronchial hyperresponsiveness, which is prevalent in patients with asthma and COPD. Although most of the research on the clinical application of FOT/IOS, mainly focuses on asthma and large airways, where FOT/IOS analysis shows its usefulness in detecting small airways for COPD identification depending on the frequency at which impedance is measured [67,68,69]. Additionally, applying machine learning to interpret FOT/IOS data after training the program with FOT/IOS data from smokers, COPD patients, and normal healthy subjects can further help improve the quantitative evaluation capability. As a proof of concept, this robust application of machine learning algorithms was found to be effective in detecting pulmonary changes with high degrees of specificity and sensitivity in various studies, showing its ability to detect subtle changes in lung function for early COPD detection [63,70]. Moreover, FOT/IOS does not require the subject to perform breathing maneuvers for measurements and only requires minimum compliance of the subject, thus making FOT/IOS useful over spirometry in a setting where patient compliance is limited, such as young children, subjects with chronic illnesses, or the elderly [71,72].

However, FOT has certain limitations as well, one example being that measurements can be affected by extra-thoracic upper airway artefacts that may distort the results when identifying small airway changes and COPD [73], thus preventing the accurate quantification of lung function and structural changes. Additionally, FOT cannot classify the causes of hyperresponsiveness, as other pulmonary deficiencies, such as asthma, may be responsible for functional change, necessitating other diagnostic tools or concurrent use of bronchodilators to confirm or validate the presence of COPD [74]. FOT/IOS is also limited in its ability to quantify regional lung function changes, as it only gauges the global impedance and hyperresponsiveness of the lung, rather than specific regions. In addition, one limitation specific to IOS is that the impulse pressure may be too intense as compared to the sequential waves of the FOT, making the patient uncomfortable [65]. 

### 4.3. Photoacoustic and Ultrasound Tomography as Emerging Lung Imaging Modalities

Another emerging diagnostic tool for COPD diagnosis is PAT, which serves as an improved alternative to the conventional CT scans that utilize ionizing radiation. Alternatively, low dose CT can reduce the risk of radiation, but it comes at the cost of decreased resolution. However, PAT uses signals from optical absorption to generate high-resolution images by exciting endogenous chromophores or exogenous contrast agents with laser beams, causing them to absorb the optical energy and increase in temperature, thus resulting in tissue expansion and, consequently, generating an ultrasound signal. This signal is then reconstructed using an algorithm to create an image that captures details of the airway and damage to lung tissue [75,76,77,78] that can then be analyzed for detection of COPD or other pulmonary disease [79]. This technique allows for improved spatial resolution, which is often limited in other optical imaging modalities due to light diffusion. Additionally, PAT does not contain optical contrasts or interfering speckle artefacts that are present in other ultrasonic imaging modalities. Moreover, PAT uses nonionizing radiation, making it a healthier alternative to modalities that utilize harmful ionizing radiation [75,80]. Nevertheless, PAT’s imaging capabilities are limited, as its imaging depth is dependent on the limit of attenuation caused by tissue [81]. PAT is further limited by its extended imaging times, which are restricted by the pulse repetition rate of the laser beams in optical excitation [75,82]. Additionally, like classical imaging modalities, PAT infers functional changes based on structural defects in the airway.

UCT is another emerging tomographic imaging modality that generates images by transmitting ultrasound waves into the tissue, which distort the waves before they are recorded by ultrasound transducers. Because UCT uses ultrasound waves instead of radiation or magnets, it can record properties of the sample that other imaging modalities are incapable of measuring, such as the attenuation of sound waves [83,84]. Additionally, UCT does not expose the subject to harmful ionizing radiation, making it a much healthier imaging modality than traditional radiation utilizing modalities. Despite its potential, UCT has not been used widely in pulmonary imaging, and its prospective application into the diagnostic field requires significant development of robust analysis tools to avoid high signal to noise ratios and to improve the quality of resolution. Overall, its soft-tissue imaging properties and innocuous imaging techniques show potential, yet are significantly limited, in capability for future-routine use lung disease diagnostics, as similar to current SOC diagnostic PFT, current prototypes cannot quantify regional or local function changes for capturing early or subtle lung function changes.

### 4.4. Electrical Impedance Tomography as Novel Diagnostics for Regional Lung Function Analysis

EIT is a promising emerging diagnostic tool that can quantify regional changes in the lung function with basic assessment and quantification of the changes in structure of the lung by non-invasively generating cross-sectional images through alternating low-dose current injections at a specific frequency via surface electrodes while measuring the changes in conductivity. This technology takes advantage of the fact that regions of muscle and blood have lower impedance than areas of fat, bone, air, and lung tissue due to free ion content, thus allowing for a ring of electrodes, generally 16 or 32, placed around the 4th and 5th intercostal space to measure impedance differences in regions of the lung, spanning all lobes. Moreover, conductivity changes in V and Q can then be reconstructed by an algorithm utilizing impedance measurements to produce images that can be used to evaluate COPD and quantify changes in lung function through comprehensive assessment of the V/Q maps. This data can be analyzed using novel quantification software(s), algorithms, and AI tools to identify specific changes in air or blood flow.

**Figure 2:**
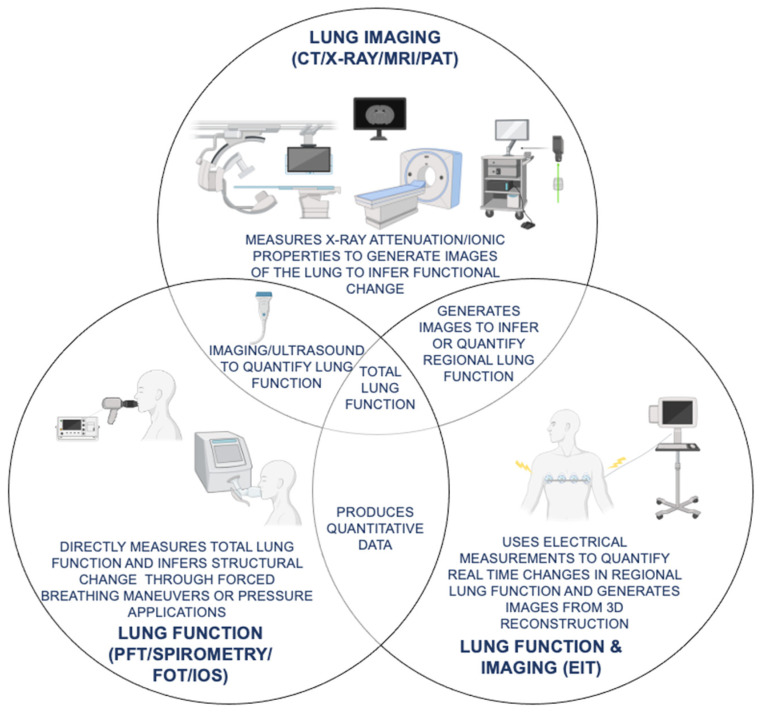
Comparison of current lung imaging and lung function tests with emerging oscillation and tomography techniques for COPD (chronic obstructive pulmonary disease) diagnosis and monitoring. Functional similarities and differences in SOC (standard of care) lung imaging (CT (computed tomography), X-ray, MRI (magnetic resonance imaging)) and lung function (PFT (pulmonary function test), spirometry, FOT (forced oscillation technique), IOS (impulse oscillometry system)) tests as compared to emerging novel modalities with both lung function and imaging capabilities (EIT (electrical impedance tomography), UCT (ultrasound computed tomography), PAT (photoacoustic tomography)) for COPD diagnosis and monitoring are shown.

One method in which EIT has been used to detect COPD is by calculating the global heterogeneity Index (HI) [85,86] that is derived from the EIT V/Q or airflow (FEV_1_/FVC) heat maps collected during tidal breathing, allowing quantification of both ventilation heterogeneity and specific assessment of lung function. Briefly, comparison of HI of COPD and non-COPD groups revealed that the non-COPD group consistently had a lower global HI than the COPD group, demonstrating that EIT is capable of both distinguishing and identifying COPD. Furthermore, ventilation heterogeneity can be used to monitor COPD by utilizing inspiratory peaks and expiratory troughs from EIT measurements to plot heating and cooling maps of expiratory time (t_E_), phase shift (PHASE), and amplitude of impedance signal (AMP), in which the heat intensity maps can be utilized to demonstrate their corresponding HIs. Assessing this metric’s efficacy in COPD recognition showed that those with COPD had an overall increased ventilation heterogeneity and coefficient of variation [85], thus showing this tool’s capability in observing changes in lung function over a period of time to quantify pulmonary diseases progression. Furthermore, reconstruction of EIT data is used to calculate regional or local FEV_1_/FVC ratios by measuring impedance values during different time points in an inspiration/expiration maneuver, further showing its potential in providing standard output measures with regional assessment capability [87]. It is noteworthy that EIT is a safer and more robust alternative to traditional SOC modalities (as shown in Figure 2) for assessing regional lung function changes, as it does not expose the patient to harmful radiation or toxic chemicals or contrast agents, thus allowing continuous real time assessment of regional lung function for monitoring disease progression at the bedside or at POC. 

In initial prototypes, EIT’s application as a diagnostic and monitoring tool was limited by its relatively low spatial resolution, as reconstructed images are not as detailed as those generated in traditional imaging modalities. However, EIT retains a high temporal resolution and quantitative assessment capabilities, making it a useful medium for monitoring regional changes [88] as compared to FOT/IOS, PAT, UCT, XPC, etc. Additionally, impedance measurements are very sensitive, capable of capturing subtle or narrow changes [89], necessitating the need for automation to minimize run-to-run variability. Moreover, currently available clinical EIT prototypes only measure cross-sections and, therefore, do not reflect conductivity measurements of regions of the lung in the z-axis. However, this limitation can be addressed easily by utilizing electrode devices designed for 3D imaging to reconstruct corresponding cross-sections of the lung and measuring it relative to time, allowing quantitative assessment of regional lung function changes.

## 5. Perspective

COPD is a progressive lung disease described as accelerated lung aging [90]. The ageing, in addition to environmental exposures, increase inflammatory–oxidative stress and cellular senescence [91,92], resulting in irreversible lung disease progression from mild to severe emphysema. However, implementing real-time lung health monitoring diagnostics along with corresponding timely interventions can decelerate disease progression to severe emphysema and prevent lung function impairment to fatal stages. Despite the prevalence of COPD globally, current SOC diagnostics have major weaknesses that limit timely treatment, as summarized in Table 2 and discussed below.

Briefly, one of the apparent limitations of current SOC diagnostics is that they are generally incapable of detecting COPD in early to mild stages, leading to the significant underdiagnosis of COPD in the population. Spirometry/PFT, ABG analysis, and DLCO are proficient in diagnosing COPD at severe stages but lack the capability to detect minute changes to allow diagnosis of pulmonary disease at the onset. Although traditional lung imaging modalities, such as CT scans, can capture structural details, which can be used to infer changes in pulmonary function that are indicative of COPD prognosis, their routine use is limited due to radiation risk. In addition, structural changes used to draw inferences, either manually by a radiologist, or utilization of quantitative software that evaluates changes in pulmonary function, are limited in their ability to detect early subtle changes with high level of confidence or consistency. In contrast, emerging diagnostic techniques, such as FOT/IOS and EIT, can detect and quantify subtle lung function defects with added robustness when used alongside corresponding AI and analysis software.

Additionally, there are a multitude of factors that cause the disproportionate number of undiagnosed patients, a main cause being that people are typically not screened for COPD unless they exhibit significant respiratory symptoms, a stage at which the disease has typically already progressed to severe stages. Instead of waiting for symptoms to present themselves, it is believed that implementing widespread screening tests for those who are at risk (e.g., smokers, >40 years old, etc.) can effectively decrease, if not eliminate, the number of subjects who are undiagnosed using current SOC, namely PFT. As a proof of concept, when a medical care system incorporates an automatic COPD risk screening in their online healthcare system, it allows for early diagnosis. Thus, when a patient is added to the system, whether they have COPD or other diseases, their characteristics, such as age and smoking status, are automatically assessed for COPD risk [93]. Furthermore, it is also critical to monitor disease progression in subjects that are already diagnosed with COPD by finding indicators predictive of acute or recurring exacerbations that will ultimately lead to steep lung function decline. By detecting these indicators early, targeted treatment measures can be implemented to reduce the impact of damage to lung structure and mechanics [94]. Moreover, there is a need for diagnostic tools to conveniently, yet accurately, screen for early indicators of COPD in undiagnosed patients as well as assess potential forthcoming exacerbations in diagnosed patients for monitoring COPD progression by robust POC and home-based lung health monitoring. Unlike non-POC diagnostic tests, which send patient samples to laboratories for analysis, taking extended periods of time, POC tests are diagnostics performed at the time and place of patient care, allowing for quick results that appropriately guide the treatment process as soon as possible. Furthermore, if the diagnostic can be exported to a home-based setting, it will be even more convenient and easy to monitor the status of the disease progression, allowing predictions of respiratory exacerbations.

The other limitation of current COPD SOC diagnostics is that they generally cannot quantify subtle COPD-induced changes in local or regional lung, preventing its utility in real-time monitoring for targeted interventions that are critical for preventing disease progression. Spirometry/PFT, ABG analysis, and DLCO device outputs are based on the overall function of the lung and therefore cannot quantify specific regional differences in the alveolar structure or function. Traditional imaging modalities, such as X-rays, CT scans, and MRIs, can monitor regional changes, but they utilize ionizing radiation or contrast agents, limiting their utility for real-time bedside monitoring of disease progression. However, emerging lung health monitoring tools, such as EIT, FOT, and IOS, can evaluate lung function in real-time and potentially allow continuous, noninvasive, bedside, and/or POC assessment once implemented. 

Additionally, the development of novel real-time COPD monitoring tools is paving the path for the use of companion diagnostics (CDx) for COPD treatment. Briefly, CDx is the concurrent use of a diagnostic test with therapeutics to observe if the administered treatment is effective in specific subject, since its efficacy varies on a patient-to-patient basis [95,96]. As a proof of concept, CDx has been utilized in multiple cancer therapies, one example being trastuzumab, which is a drug that targets the receptor tyrosine-protein kinase (HER2) often overexpressed in subjects with breast cancer. By adjusting levels of trastuzumab administered depending on the response of the patient to the drug, physicians were able to find optimal treatment parameters while also quantifying the levels of HER2 present [97,98]. In contrast, personalized treatment of COPD requires further clinical development, where several prognostic and inflammatory biomarkers have shown promise as CDx utility. As an example, during exacerbations, the eosinophilic airway is often inflamed, resulting in an increase in eosinophil levels. CDx is implemented by using the levels of eosinophils to guide corticosteroid use with the goal of reducing exacerbations [99,100].

However, the utility of CDx in COPD treatment is currently limited by the lack of appropriate diagnostic and monitoring tools, highlighting the unmet need for a SOC real-time bedside lung function monitoring device or a home-based or POC prognostic test. Moreover, CDx can be further specialized for tracking local or regional pulmonary function changes for real-time assessment of efficacy of the intervention. As discussed, current lung imaging modalities allow lung function quantification but do so indirectly by drawing inferences from structural changes, instead of quantifying dynamic changes in the lung function. As an example, images generated from CT scans can be used to infer changes in pulmonary function based on parametric response or flow rate analysis of CT data to evaluate changes in lung density, V/Q, or other lung function outcome measures to infer presence of enlarged airspaces, obstruction, lung tissue destruction, etc., for predicting lung function instead of directly quantifying changes in lung dynamics [101,102]. In contrast, EIT is emerging as a powerful diagnostic tool that directly monitors subtle regional lung function changes in real-time without radiation risk or significant discomfort to the subject. Briefly, it can provide direct assessment of real-time changes in lung function in various regions through voxel-by-voxel segmentation and 3D reconstruction of impedance data for quantifying dynamic and subtle changes in air or blood flow for providing quantitative assessment of conventional lung function outcome measures as a practical solution over lung imaging modalities. Additionally, EIT is easily accessible as a bedside monitoring CDx, utilizing clinically available EIT devices, such as Dräger’s PulmoVista^R^ 500 and Swisstom AG’s Swisstom BB^2^, etc., which only require a sensor belt with electrodes and a computer or tablet with software for impedance measurements. Moreover, for patient comfort, commercial EIT vests have been designed to conveniently strap to the subject in seconds with no requirement of patient compliance as in conventional PFT measurements, thus allowing bedside or real-time monitoring of lung and/or heart function [103]. Moreover, EIT can significantly benefit from emerging AI and deep learning tools to further improve automation and resolution for a user-friendly diagnostic solution.

In summary, the implementation of POC tests that can be performed quickly and conveniently at the site of care or at home will be the key to early diagnosis of COPD for identifying the large number of subjects with COPD that are currently undiagnosed. Moreover, this will allow early-intervention before disease progression to severe stages, thus improving the health of millions while reducing health care costs by improving both QoC and QoL. Additionally, novel lung health monitoring tools will be vital in the implementation of CDx to directly detect subtle regional lung function changes or prognosis, thereby validating the efficacy of specific treatments in COPD subjects and providing tailored or targeted intervention for delivering on the promise of precision medicine. Thus, with this added utility, timely intervention and tailored therapy, we will have better patient outcomes, significantly reducing COPD associated mortality.

## Figures and Tables

**Table 1:** Comparative analysis of current standard of care and emerging COPD diagnostic and monitoring tools for evaluating regional lung function, providing quantitative output, potential utility as a CDx, known capability for exacerbation detection, available diagnostic setting, and level of risk to the patient.

Lung Diagnostics and Monitoring	Status	Regional Lung Function	Quantitative Output	CDx Use	Early Exacerbation	Diagnostic Setting	Risks
**6MWT**	Current	No	No/Limited	Low	Limited	Bedside	No
**COPD Questionnaire**	Current	No	Yes	Low	Yes	Bedside	No
**PFT/Spirometry**	Current	No	Yes	Low	No	PFT Lab/Bedside	No
**CT/MRI**	Current	Yes/Inference	Yes/Inference	Low	No	Radiology	High
**ABG and DLCO**	Current	No	Yes	Low	No	Bedside	Minimal
**FOT/IOS**	Emerging	No	Yes	Medium	No	Bedside	Minimal
**PAT**	Emerging	Yes	Yes/Inference	Low	No	Radiology/Clinic	Minimal
**UCT/XPC**	Emerging	No	Yes/Inference	Low	No	Radiology/Clinic	Medium
**EIT**	Emerging	Yes	Yes	High	Yes	Bedside	No

Chronic obstructive pulmonary disease (COPD), PFT: pulmonary function test, 6MWT: six minute walk test, ABG/DLCO: arterial blood gas/diffusing capacity for carbon monoxide, CT: computed tomography; MRI: magnetic resonance imaging; FOT: forced oscillation technique, IOS: impulse oscillometry system, EIT: electrical impedance tomography, XPC: X-ray phase contrast, UCT: ultrasound computed tomography, PAT: photoacoustic tomography, CDx: companion diagnostics.

**Table 2:** Scope, risks, and/or limitations of current and emerging chronic obstructive pulmonary disease diagnostics and monitoring techniques.

COPD Diagnostic Comparison	Disease Details	Diagnostic Accuracy	Risks	Patient Compliance	Time
**X-ray/XPC**	Low	Low	Low radiation	No	15 min
**CT/MRI**	High	High	Radiation/Contrast Agents	No	20 min–2 h
**PFT/Spirometry**	Low	Moderate	Low	Yes	30 min–1 h
**ABG/DLCO**	Low	Moderate	Low	No	15 min
**FOT/IOS**	Low	Moderate	NA	No	Real Time
**PAT**	Moderate	Moderate	NA	No	Real time
**UCT**	Low	Moderate	Low	No	Real time
**EIT**	Moderate	High	NA	No	Real Time

MRI: magnetic resonance imaging, CT: computed tomography, PFT: pulmonary function test, ABG/DLCO: arterial blood gas/diffusing capacity for carbon monoxide, FOT: forced oscillation technique, IOS: impulse oscillometry system, XPC: X-ray phase contrast, PAT: photoacoustic tomography, UCT: ultrasound computed tomography, EIT: electrical impedance tomography.

## Data Availability

Not Applicable.
